# Location Matters: Trends in Inequalities in Child Mortality in Indonesia. Evidence from Repeated Cross-Sectional Surveys

**DOI:** 10.1371/journal.pone.0103597

**Published:** 2014-07-25

**Authors:** Andrew Hodge, Sonja Firth, Tiara Marthias, Eliana Jimenez-Soto

**Affiliations:** 1 School of Population Health, The University of Queensland, Brisbane, Queensland, Australia; 2 Center for Health Policy and Management, Faculty of Medicine, Gadjah Mada University, Yogyakarta, Indonesia; Medical College of Soochow University, China

## Abstract

**Background:**

Considerable improvements in life expectancy and other human development indicators in Indonesia are thought to mask considerable disparities between populations in the country. We examine the existence and extent of these disparities by measuring trends and inequalities in the under-five mortality rate and neonatal mortality rate across wealth, education and geography.

**Methodology:**

Using data from seven waves of the Indonesian Demographic and Health Surveys, direct estimates of under-five and neonatal mortality rates were generated for 1980–2011. Absolute and relative inequalities were measured by rate differences and ratios, and where possible, slope and relative indices of inequality. Disparities were assessed by levels of rural/urban location, island groups, maternal education and household wealth.

**Findings:**

Declines in national rates of under-five and neonatal mortality have accorded with reductions of absolute inequalities in clusters stratified by wealth, maternal education and rural/urban location. Across these groups, relative inequalities have generally stabilised, with possible increases with respect to mortality across wealth subpopulations. Both relative and absolute inequalities in rates of under-five and neonatal mortality stratified by island divisions have widened.

**Conclusion:**

Indonesia has made considerable gains in reducing under-five and neonatal mortality at a national level, with the largest reductions happening before the Asian financial crisis (1997–98) and decentralisation (2000). Hasty implementation of decentralisation reforms may have contributed to a slowdown in mortality rate reduction thereafter. Widening inequities between the most developed provinces of Java-Bali and those of other island groupings should be of particular concern for a country embarking on an ambitious plan for universal health coverage by 2019. A focus on addressing the key supply side barriers to accessing health care and on the social determinants of health in remote and disadvantaged regions will be essential for this plan to be realised.

## Introduction

In the Asia-Pacific region, large and populous countries like Indonesia are experiencing a profound transformation. At the beginning of the 1970s, Indonesia was one of the poorest countries in the world with low literacy and life expectancy rates. It is now classified as a middle-income country with 92 percent of the population being literate and life expectancy standing over 70 years [1]. However, concerns remain about stagnating progress and widening inequities. Previous studies have shown that access to health facilities and services is higher for those in urban, educated and wealthier households [2]–[4]. Health insurance coverage is low among the poor and near-poor [5]. Urban households on average enjoy better access to clean water and sanitation and facility-based delivery [3], [6]. Higher level of maternal education is associated with higher utilisation of antenatal care services in Indonesia [7] as well as with lower early neonatal death [8]. Immunisation rates are higher for children in educated households [9]. Wealth related inequality is large, particularly for interventions that might attract out-of-pocket expenses, such as facility-based delivery or skilled birth attendance [10]. These disparities present serious challenges to the government's goal of achieving universal health coverage by 2019. They are compounded by the difficulties of delivering services in a diverse and large archipelago with more than 17,000 islands. When compared to the most developed provinces of Java-Bali, other regions are particularly disadvantaged in terms of social determinants of health, infrastructure, delivery of services and human resources [3], [11], [12].

In this paper we focus on trends and inequalities of under-five mortality rate (U5MR) and neonatal mortality rate (NMR). We examine the extent to which national progress masks disparities across a range of equity markers. We analyse both absolute and relative inequalities for U5MR and NMR across wealth, education and geography (urban/rural and island groups). A previous study examined trends in relative inequalities for U5MR during 1982–1997 using similar equity markers to those proposed in this study [13]. The authors found that during this period characterised by remarkable economic growth, mortality rates declined substantially, but were not accompanied by increasing relative inequalities across the socio-economic spectrum as originally predicted [13]. Our analysis shows that relative inequalities are only one side of the story and in line with the most recent literature [14]–[16] examine both absolute and relative measures of inequality. This allows more robust conclusions regarding the extent of inequality. As recently documented, results on the extent to which inequalities show an increasing or decreasing trend over time are strongly influenced by the choice of absolute or relative measures [17].

## Methods

### Ethics statement

The datasets used in this study were obtained from the MEASURE DHS website, <http://www.dhsprogram.com>. Full review of this study from an institutional review board was not sought as this manuscript involved secondary data analysis of datasets that are publicly available, anonymous, with no identifiable information on the survey participants.

### Data

For this study, analyses are based on survey data from the Indonesian Demographic Health Surveys (DHS). The DHS are repeated cross-sectional surveys undertaken by Statistics Indonesia (Badan Pusat Statistik – BPS) in collaboration with the worldwide DHS program. The surveys are designed to collect demographic, socioeconomic and health data, including information on fertility, and maternal and child health. The surveys are representative at the national and provincial levels, except the first wave which was representative of 93% of the total population. Further details on data collection, sample design and management procedures are described elsewhere [3], [18]–[23]. We utilised seven survey waves: 1987, 1991, 1994, 1997, 2002–03, 2007–08 and 2012. The corresponding households (women aged 15–49) samples were: 14,142 (11,884); 26,858 (22,909); 33,738 (28,168); 34,255 (28,810); 33,088 (29,483); 40,701 (32,895); and 43,853 (45,607), respectively.

We assembled records on every child ever borne to female respondents from the complete birth history (CBH) modules as inputs into the mortality estimation. The combined dataset contained 530,437 children ever borne under the age of five. The datasets were cleaned by deleting duplicates and children who had unfeasible birth dates and death ages were omitted (e.g. children recorded to have died after the date of a survey interview).

Disparities in child mortality were assessed across four equity markers. Socioeconomic position was measured by wealth and the mothers' level of education. A wealth index [24] was supplied only from the 1997 survey wave. Hence, we constructed an index using principal components analysis for each survey wave to maintain a consistent approach [25]. Given the absence of income and expenditure data, an assets-based measure was constructed using data on a household's ownership of assets such as housing materials, ownership of durable goods, and access to improved water and sanitation. Our index was highly correlated (>0.90) with the DHS wealth index for waves it was available. The index ranged between −9.1 to 8.4 and ranked households into wealth quintiles. In terms of maternal education, we categorised mothers as having attained no education (0 years of schooling), some primary education (1–6 years), completed primary education (7 years) or some secondary or higher education (>7 years). We tested the robustness of the results to changes in wealth and education classifications, with wealth grouped into low, middle and high income subpopulations and maternal education classed alternatively as no, primary, secondary and higher education, or no, incomplete primary, complete primary, incomplete secondary, complete secondary and higher education. Data on wealth was complete for all households, while maternal education was missing for only 3 women in the 2007 wave.

Geographical-based disparities were measured using rural-urban location and grouping the country islands into the usual classification of Java-Bali, Sumatra and the rest, which comprises the areas of Kalimantan, Sulawesi, Nusa Tenggara, Maluku and Papua. Java-Bali region is the most developed part of Indonesia, having significantly more health providers, higher population density [26], [27] as well as higher utilisation of maternal health services and lower neonatal mortality risks [8]. After Java-Bali, Sumatera region is the next better-off part of Indonesia, followed with the less developed areas that consists of Kalimantan, Sulawesi, Nusa Tenggara, Maluku and Papua.

### Statistical analyses

Under-five and neonatal mortality, and associated 95% confidence intervals, at both national and sub-national levels, were estimated directly using CBHs, adhering to the methods of Rajaratnam and colleagues [28]. Due to the relative rarity of child deaths, mortality rates were estimated biennially. The two-year estimates were created by pooling data across all the surveys and structuring the data into person-months by detailing for a five year period each child's life or death in each month. Mortality rates were computed from age-group mean survival probabilities and the associated survival rates, accounting for sample weights.

Both absolute and relative measures of inequalities were computed given the debate over the distinction between relative and absolute scales and their interpretation [17], [29], [30]. The following four measures were computed: rate difference (RD) and the slope index of inequality (SII) to capture absolute inequalities, and rate ratio (RR) and the relative index of inequality (RII) to gauge relative inequalities [29]. RDs and RRs were computed for each sub-population in reference to a base group. For wealth and education the highest ranked socioeconomic group was chosen as the base, while for the geography-related equity markers, the category with the lowest average under-five mortality rate over the sample period was chosen as the referent. We report RDs and RRs for wealth and education as comparisons between the lowest and highest socioeconomic groups (i.e. lowest vs. highest wealth quintile and no education vs. some secondary or higher education). The results for the other group comparisons are available upon request.

The merits of the RIIs and SIIs over the RDs and RRs are well known [31]. In particular, these indexes account for any changes in the distribution of the equity marker. Unfortunately, the need for ordinal groups to rank the population implies that the RIIs and SIIs are only feasible for mortality rates stratified by wealth and education. The RIIs and SIIs were estimated via weighted linear regression of the mortality rates on the midpoint of the cumulative population distribution ranked by the socioeconomic indicator [32].

Confidence intervals on the mortality estimates and on the corresponding RDs and RRs were constructed by generating 1,000 simulations of the survival probability for each time-period/age-group assuming a binomial distribution, where the probability equals the mean survival probability and sample size is the number of person-months observed in the time-period/age-category. The mortality rate was then calculated for each time-period in each simulation. This process was undertaken for each group of each equity marker and the RDs and RRs computed in each simulation. The 2.5^th^ and 97.5^th^ percentiles were then extracted as the lower and upper confidence bounds on the mortality, RD and RR estimates for each time-period. For the RIIs and SIIs, the 95% confidence intervals were calculated using standard methods discussed by Hayes and Berry [33].

Changes in disparities over time were gauged via comparisons of the mortality rates and measures of inequalities (and the associated uncertainty estimates) over the sample period and tests of the statistical significance of a linear trend in these estimates [31]. Given the possibility of correlation in the regression error terms applied to time series data, we used Newey-West standard errors (using one lag) in these regressions, which are robust to both heteroskedasticity and serial correlation [34]. In the cases of RRs and RIIs, we used the natural logarithm of these measures in the regressions and we report the exponentiated trend coefficients, which can be interpreted as the average ratio change in RR or RII per period. For RDs and SIIs, we report the trend coefficient, which can be interpreted as the average absolute change per period in the inequality measures over the sample.

All statistical analyses were conducted using the two software programs, *Stata* and *R*.

## Results

**Figure 1:**
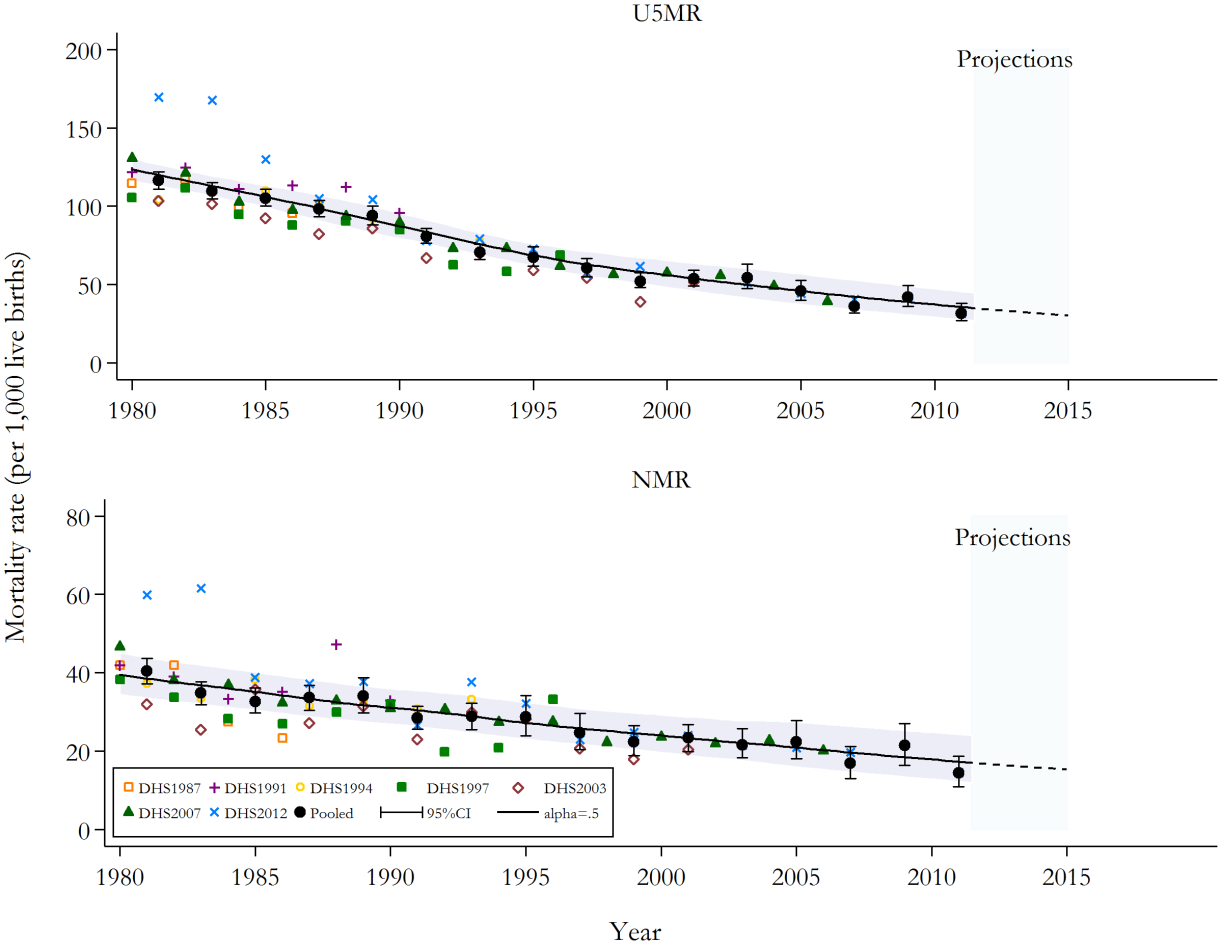
Under-five and neonatal mortality rates (per 1,000 live births) at the national level: actual 1980–2011; projected to 2015. National estimates by source and using the pooled data are displayed. Loess regression using a smoothing parameter of 0.5 was applied to produce the continuous series [51]. The last set of parameter estimates for the Loess regression were utilised to project mortality rates toward 2015. The solid and semi-broken lines represent the continuous mortality estimates calculated from the two-year estimates, while the shaded area signifies the corresponding 95% confidence intervals. U5MR, under-five mortality rate; NMR, neonatal mortality rate; DHS, Demographic Health Survey; CI, confidence intervals.

Nationally, the U5MR and NMR have declined since 1980. Figure 1 presents national estimates. The U5MR has fallen from 116 (95% CI 111 to 122) deaths per 1,000 live births in 1980–91 to 31 (95% CI 27 to 38) in 2010–11. The rates of reduction in the NMR were lower but nonetheless considerable, declining from 40 (95% CI 37 to 44) in 1980–81 to 14 (95% CI 11 to 19) in 2010–11. Closer examination of trends in disparities showed that these reductions at the national level masked significant within-country inequalities.

**Figure 2:**
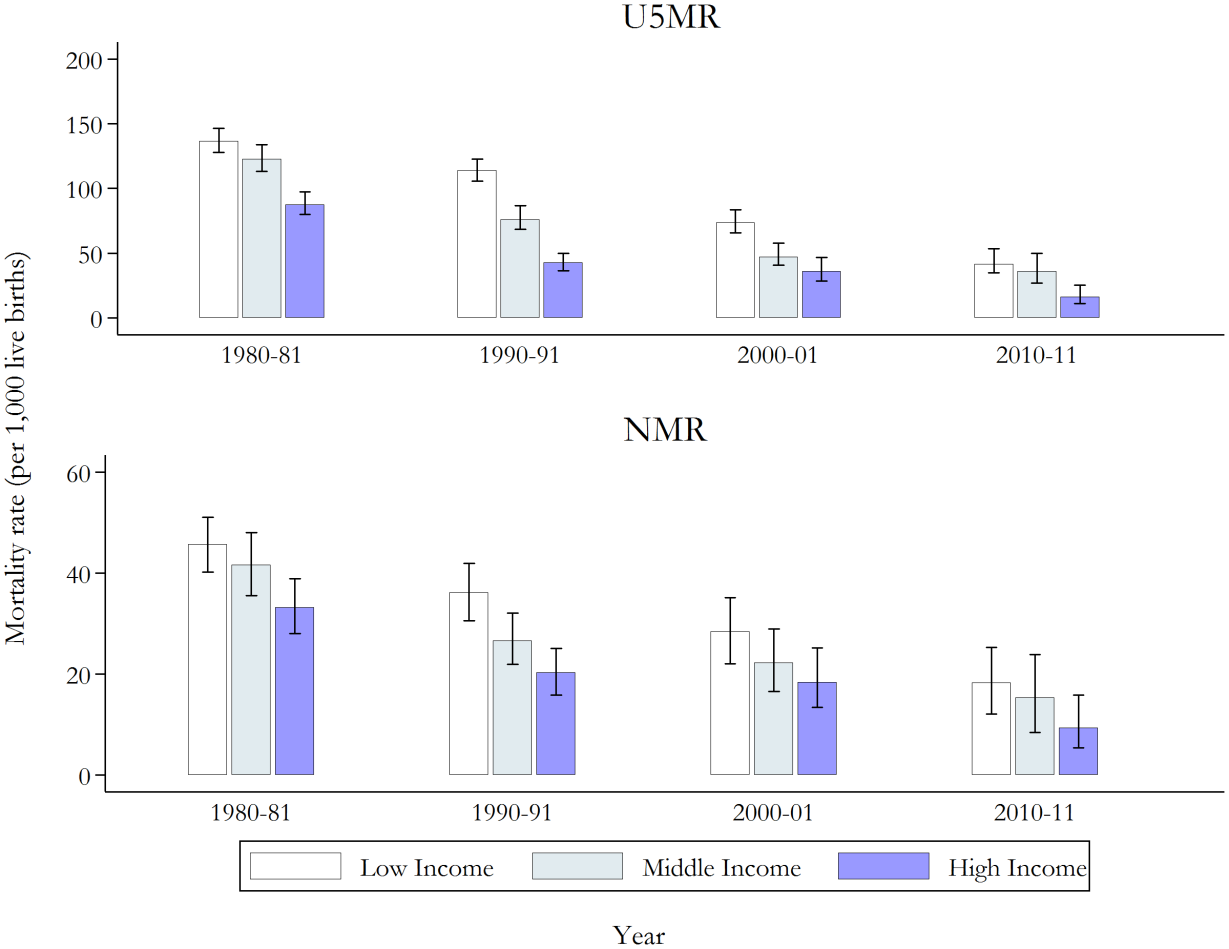
Under-five and neonatal mortality rates (per 1,000 live births) by wealth groups for selected two-year periods, with 95% confidence intervals. See Tables S3 and S4 in File S1 for full results. The population was divided into thirds using the wealth index. U5MR, under-five mortality rate; NMR, neonatal mortality rate.

As illustrated in Figure 2, high income households experience lower rates of under-five and neonatal mortality compared to households with poorer socioeconomic status. However, the gap has decreased over time. This pattern is confirmed by the estimates in Table 1, which presents measures of absolute (i.e. RD and SII) and relative (i.e. RR and RII) inequalities across wealth and educational clusters. The estimates suggest that absolute inequalities in under-five and neonatal mortality have decreased, while relative inequalities have stabilised or possibly increased. For example, the RD and SII for under-five mortality reduced from 74 (95% CI 59 to 90) in 1980–81 to 29 (95% CI 8 to 46) in 2010–11 and from 87 (95% CI 39 to 134) in 1980–81 to 40 (95% CI −10 to 90) in 2010–11, respectively. While the RRs and RIIs for the same two-year periods increased from 2.03 (95% CI 1.7 to 2.4) to 2.33 (95% CI 1.2 to 3.99) and from 2.19 (95% CI 1.1 to 3.3) to 3.92 (95% CI −5.6 to 13.4), respectively. Positive trends in RIIs are associated with greater statistical significance than the trends in rate ratios, which is to be expected as the RII incorporates changes in the underlying income and education distributions over the sample period. It is also evident that the decline of absolute inequalities over time is less statistical significant for neonatal than under-five mortality. Similar patterns (results not reported) were observed when using the survey-supplied wealth index and when wealth and education clustered were re-categorised.

**Table 1:** Inequalities in under-five and neonatal mortality by wealth and education markers for selected years, with 95% confidence intervals and *p*-values for trend.

Equity Marker	Relative Inequalities				Absolute Inequalities			
	RR	(95% CI)	RII	(95% CI)	RD	(95% CI)	SII	(95% CI)
**Wealth**								
*U5MR*								
1980–81	2.03	(1.73; 2.36)	2.19	(1.12; 3.25)	74.3	(59; 89.4)	86.95	(39.1; 134.8)
1990–91	3.52	(2.8; 4.43)	5.68	(3.54; 7.82)	91.1	(78.3; 104.4)	117.59	(102.62; 132.56)
2000–01	3.07	(2.18; 4.16)	3.98	(0.44; 7.52)	55.7	(41.4; 69)	67.03	(36.68; 97.37)
2010–11	2.33	(1.24; 3.99)	3.92	(−5.58; 13.41)	29.2	(8.4; 46.1)	40.26	(−10.32; 90.85)
Trend^1^ [*p*-value]	1.008	[0.641]	1.039	[0.061]	−3.531	[0.000]	−3.692	[0.003]
Trend^2^ [*p*-value]	1.034	[0.019]	1.065	[0.005]	−2.857	[0.003]	−3.014	[0.018]
*NMR*								
1980–81	1.57	(1.21; 2.05)	1.72	(0.99; 2.44)	17.9	(8; 27.9)	21.37	(5.57; 37.16)
1990–91	2.22	(1.56; 3.28)	2.71	(1.55; 3.87)	21.9	(12.9; 31.4)	26.88	(17.4; 36.36)
2000–01	2.27	(1.42; 3.65)	2.56	(−2.03; 7.14)	19.6	(8.4; 30)	21.22	(−12.88; 55.31)
2010–11	2.01	(0.86; 4.76)	3.19	(−1.7; 8.07)	11.3	(−2.6; 23.5)	16.12	(−0.38; 32.62)
Trend^1^ [*p*-value]	1.019	[0.111]	1.033	[0.001]	−0.398	[0.031]	−0.378	[0.142]
Trend^2^ [*p*-value]	1.034	[0.029]	1.037	[0.019]	−0.243	[0.141]	−0.329	[0.264]
**Education**								
*U5MR*								
1980–81	2.47	(2.06; 2.91)	2.67	(0.32; 5.01)	90.4	(74.3; 106.4)	103.75	(26.7; 180.81)
1990–91	2.82	(2.3; 3.43)	4.11	(−2.52; 10.75)	88.5	(70.1; 109.4)	98.02	(20.54; 175.49)
2000–01	2.14	(1.46; 3.01)	3.78	(−6.01; 13.57)	46.6	(21.6; 78.2)	63.38	(−25.85; 152.61)
2010–11	3.36	(2.27; 6.5)	5.47	(−31.66; 42.61)	62.1	(37.6; 135)	45.43	(−65.06; 155.92)
Trend^1^ [*p*-value]	1.003	[0.783]	1.053	[0.001]	−2.977	[0.002]	−4.450	[0.000]
Trend^2^ [*p*-value]	0.989	[0.151]	1.303	[0.052]	−4.209	[0.000]	−4.969	[0.000]
*NMR*								
1980–81	1.78	(1.37; 2.32)	1.87	(0.87; 2.88)	21.5	(12; 31)	24.21	(5.02; 43.41)
1990–91	2.29	(1.55; 3.27)	2.78	(−3.15; 8.7)	27.8	(14.1; 42.1)	27.30	(−19.27; 73.88)
2000–01	2.38	(1.16; 4.04)	3.09	(−7.96; 14.14)	27.3	(3.9; 54.5)	24.70	(−36.82; 86.22)
2010–11	1.10	(0.43; 3.74)	3.50	(−15.18; 22.19)	1.2	(−8.5; 29.5)	16.66	(−36.69; 70.02)
Trend^1^ [*p*-value]	0.971	[0.023]	1.041	[0.001]	−1.028	[0.004]	−0.356	[0.055]
Trend^2^ [*p*-value]	0.973	[0.230]	1.042	[0.049]	−0.933	[0.102]	−0.386	[0.210]

*Notes*: See File S1 for full results. U5MR, under-five mortality rate; NMR, neonatal mortality rate; CI, confidence interval; RR, rate ratio; RD, rate difference; RII, relative index of inequality; SII, slope index of inequality. The small number of observations and possible non-linear relationships implies that the trend estimates should be treated with caution. Additionally, since the bounds of the CI depend on the mean of mortality, comparisons over time must be treated cautiously.

1 Trend regressions with all observations included.

2 Trend regressions with the last 2 periods (i.e. 2008–09 and 2010–11) excluded from the sample.

A similar pattern is observed between the disparities in child mortality across rural and urban locations. As reported in Table 2, absolute disparities in both under-five and neonatal mortality were found to have reduced over time. For example, the RD for neonatal mortality reduced from 13 (95% CI 6 to 20) in 1980–81 to 5 (95% CI −2 to 13) in 2010–11. Relative inequalities showed no statistically significant trends at conventional levels, which is expected given the lower reduction in neonatal rates over the sample period.

**Table 2:** Inequalities in under-five and neonatal mortality (per 1,000 live births) by rural-urban and island division for selected years, with 95% confidence intervals and *p*-values for trend.

Equity Marker	U5MR				NMR			
	Rel. Ineq.^3^	(95% CI)	Abs. Ineq.^4^	(95% CI)	Rel. Ineq.^3^	(95% CI)	Abs. Ineq.^4^	(95% CI)
**Rural/Urban** *(base = Urban)*								
*Rural*								
1980–81	1.24	(1.12; 1.39)	24.3	(13.28; 35.71)	1.41	(1.18; 1.7)	13.0	(6.25; 19.55)
1990–91	1.75	(1.52; 2)	40.3	(31.36; 48.9)	1.90	(1.51; 2.41)	16.0	(10.64; 21.36)
2000–01	1.43	(1.14; 1.81)	18.7	(7.28; 30.11)	1.18	(0.88; 1.59)	3.7	(−2.91; 10.41)
2010–11	1.66	(1.13; 2.22)	15.5	(3.89; 25.09)	1.45	(0.86; 2.55)	5.3	(−2.16; 13.4)
Trend^1^ [*p*-value]	1.009	[0.151]	−1.377	[0.005]	0.997	[0.784]	−0.560	[0.030]
Trend^2^ [*p*-value]	1.009	[0.352]	−1.416	[0.029]	0.992	[0.600]	−0.756	[0.025]
**Island Division** *(base = Java and Bali)*								
*Sumatra*								
1980–81	0.99	(0.9; 1.09)	−0.9	(−11.7; 9.8)	0.96	(0.81; 1.15)	−1.4	(−8.26; 5.29)
1990–91	1.15	(1.01; 1.31)	11.2	(0.4; 21.5)	1.06	(0.85; 1.32)	1.6	(−5.09; 7.92)
2000–01	1.27	(1.02; 1.55)	12.0	(1.1; 22.2)	1.31	(0.98; 1.87)	6.4	(−0.53; 14.24)
2010–11	0.98	(0.7; 1.57)	−0.5	(−10.6; 13)	1.14	(0.63; 2.13)	2.0	(−6.65; 10.79)
Trend^1^ [*p*-value]	1.017	[0.133]	0.991	[0.114]	1.030	[0.002]	0.764	[0.004]
Trend^2^ [*p*-value]	1.033	[0.000]	1.767	[0.003]	1.034	[0.011]	0.904	[0.009]
*Kalimantan, Sulawesi, NTMP*								
1980–81	1.18	(1.07; 1.29)	19.8	(7.7; 31.9)	1.07	(0.91; 1.26)	2.9	(−4.14; 9.54)
1990–91	1.36	(1.2; 1.54)	26.6	(16.4; 36.9)	1.06	(0.86; 1.33)	1.7	(−4.4; 8.14)
2000–01	1.62	(1.28; 2.02)	27.5	(14.8; 41.5)	1.21	(0.85; 1.77)	4.5	(−3.76; 13.04)
2010–11	1.63	(1.15; 2.36)	17.5	(4.8; 31.1)	1.07	(0.58; 2.02)	1.0	(−7.5; 9.61)
Trend^1^ [*p*-value]	1.027	[0.002]	0.192	[0.608]	1.020	[0.052]	0.384	[0.137]
Trend^2^ [*p*-value]	1.056	[0.001]	0.727	[0.021]	1.030	[0.012]	0.609	[0.054]

*Notes*: See File S1 for full results. U5MR, under-five mortality rate; NMR, neonatal mortality rate; CI, confidence interval; Ineq., Inequalities; NTMP, Nusa Tenggara, Maluku and Papua; Rel. Ineq., relative inequality; Abs. Ineq., absolute inequality. The small number of observations and possible non-linear relationships implies that the trend estimates should be treated with caution.

1 Trend regressions with all observations included.

2 Trend regressions with last 2 periods (i.e. 2008–09 and 2010–11) excluded from the sample.

3 Rate Ratio.

4 Rate Difference.

**Figure 3:**
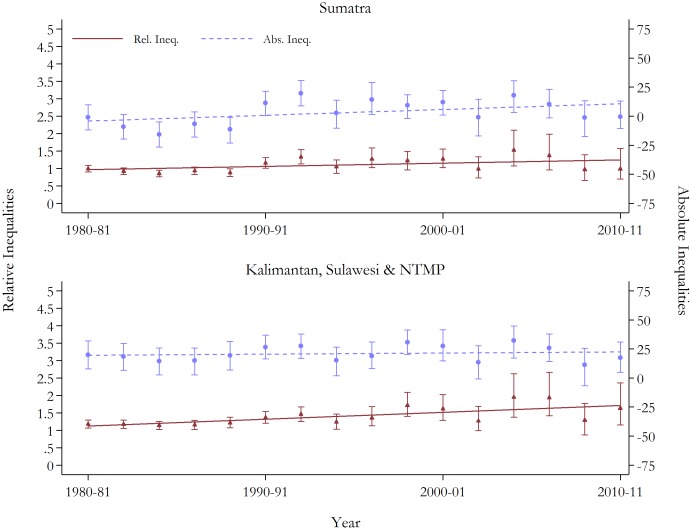
Trends in relative and absolute inequalities in under-five mortality by island groups, with 95% confidence intervals. See Table S2 in File S1 for full results. Base group is Java & Bali. Rel, Ineq., relative inequality; Abs. Ineq. Absolute inequality; NTMP, Nusa Tenggara, Maluku and Papua.

**Figure 4:**
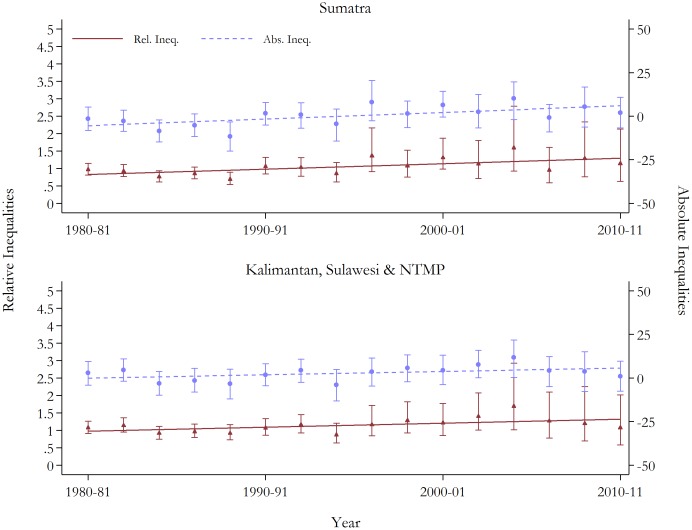
Trends in relative and absolute inequalities in neonatal mortality by island groups, with 95% confidence intervals. See Table S2 in File S1 for full results. Base group is Java & Bali. Rel, Ineq., relative inequality; Abs. Ineq. Absolute inequality; NTMP, Nusa Tenggara, Maluku and Papua.

Contrary to the general pattern, the estimates by island division showed patterns of widening inequalities on both relative and absolute scales. Upward trajectories in relative inequalities were observed across all the islands. Corresponding RDs have increased, consistent with increasing inequality; although, this absolute gap has reduced in Sumatra in the two most recent time periods. The magnitudes of rising inequalities are displayed in Figures 3 and 4. The pattern of climbing inequalities is enhanced when the two final time period estimates (i.e. 2008–09 and 2010–11) are excluded from the trend regressions. These periods rely on data from only one survey and are estimated with the fewest observations (i.e. these periods have approximately 1/3 and 2/5 fewer observations than in 2006–07, respectively), and hence, the robustness of the estimates is questionable. When removed, statistically significant positive trends at conventional levels in relative and absolute inequalities for both under-five and neonatal mortality are observed in all regions.

The full biennial mortality and absolute and relative inequality estimates are presented in Tables S1–S4 in File S1.

## Discussion

Our findings show that Indonesia has achieved considerable success in reducing U5MR at the national level. We also observe substantive improvements in NMR, which rely on the more difficult task of strengthening health systems. The largest gains for both U5MR and NMR were achieved in the earlier years that preceded the Asian financial crisis (1997–98) and decentralisation (2000). In the last decade, mortality rates have continued to decline but at lower rates, particularly for neonatal mortality which makes up an increasing proportion of under-five mortality. Similar patterns have been observed for other MDG indicators, such as poverty rates, which also show strong declines in the 1980s–1990s and steady but slower reductions in the last decade [35]. With strong rates of economic growth averaging 5.4% over the 2001–2010 period, there are no doubts that Indonesia fully recovered from the financial crisis [35], leaving decentralisation as a potential culprit for the slowdown in improving critical health and poverty indicators. In health, decentralisation was seen as a way of improving local authorities' ability to address local problems and, accompanied by a substantial increase in public spending on health, was expected to improve health system performance. Available evidence suggests that this has not been the case [36], [37]. A combination of limited local capacity [2], [36]–[38], confusion of responsibilities for different levels of government [9], [39], [40] and a complicated funding mechanism with delays in disbursement and limited discretion over resource allocation at the local level [37], [41], [42] may have contributed. Moreover, the ‘Big Bang fashion’ of implementing decentralisation in Indonesia (i.e. over less than 2 years) may be partly to blame; allowing insufficient time for lower levels of government to build their capacity or for the development of coherent and consistent operational guidelines and laws dictating the responsibilities of different levels of government [40].

Of most concern is the persistent, and sometimes increasing, inequalities observed. Disparities are strongest in relative terms, although across island divisions absolute inequalities remain and show some signs of widening. On the positive side, for U5MR absolute inequalities across wealth and education show a decreasing and statistically significant trend. This finding suggests that gains have been achieved across the spectrum and have not been concentrated only on the wealthy and educated households. However, this is tempered by the observed increase in relative inequalities for wealth and education, with the RII rising from 2.67 in 1980–1981 to 5.47 in 2010–2011. Such strong, steady and statistically significant increases suggest that those at the top have benefited proportionally more than those at the bottom of the distribution. Worryingly, for NMR, usually a good indicator of the strength of health systems, relative inequalities have been on the rise across both wealth and education with positive and statistically significant trends for RII of NMR. Unlike in the case of U5MR, the corresponding measures of absolute inequalities while declining are generally not statistically significant for NMR. This is barely surprising. Strong improvements in NMR for the most disadvantaged groups require substantial political and financial investments in addressing important health system constraints, such as distribution of human resources and lack of quality of care in both the private and the public sector.

The observed statistically significant reductions in absolute inequality for both U5MR and NMR across the urban/rural divide are more difficult to examine in a rapidly urbanising country like Indonesia [43], [44]. The results can be interpreted in a number of ways. One possibility is that rural populations are catching up with urban areas, where initial improvements in mortality are now levelling off. Complex population dynamics, including disadvantaged rural households migrating into urban slums seeking economic opportunities [45], could have also contributed to the closing gap. While the urban population continues to grow annually at 2.7% in the year 2012, a negative annual growth rate of −0.31% was observed in rural areas, suggesting an important rural-to-urban migration trend [46]. Such population dynamics have made it increasingly difficult for urban infrastructure and service provision to keep pace with demand, particularly from those living in slums and illegal settlements [47]. This situation, compounded by a burgeoning and largely unregulated private sector, has contributed to financial barriers and low quality of care in urban areas [48].

In terms of geographical markers however, for both U5MR and NMR, we observe widening absolute and relative inequalities between the prosperous Java-Bali and the most disadvantaged provinces. This is not surprising when taking into account the fact that these provinces continue to face high poverty rates, under-nutrition, low density of health workers and limited access to health facilities and services [9]. For example, a recent report found that around 25% of health centers in the country are without doctors, and most of them are located in the remote provinces [49].

Our findings for mortality indicators mirror those of a recent OECD study which examined recent poverty trends in the country [35]. The authors found that notwithstanding the rapid economic growth of the last three decades in the country, regional disparities in poverty rates still persist [35]. The widening gap in basic health indicators like U5MR and NMR are of most concern in a country like Indonesia, where decentralisation reforms were driven by political instability and several provinces demanding independence [50].

The presentation of trends in both absolute and relative inequalities drawn from large, high-quality, nationally representative datasets with few missing observations is the notable strength of this study. Two factors, however, represent important limitations. First, large uncertainty intervals are associated with some groups, especially in the last two biennial periods. Accordingly, some caution is required in asserting the degree of inequality. However, the patterns of inequalities are likely to be relatively accurate. We have tested the sensitivity of the patterns by excluding these periods and have found that this generally strengthens the results. The limited number of data points does imply that the statistical power of significance tests for linear trends are insufficient to detect small changes. Yet, we have only emphasised consistent trends, which are supported by other evidence in the literature. Second, well-known measurement errors associated with survey data and the potential influence of recall bias may have affected the results. The pooling of data from multiple surveys should help to address these potential biases and mitigate recall bias when surveys overlap.

Our study provides a robust analysis of inequality trends in U5MR and NMR for Indonesia. It shows that notwithstanding national progress made on reducing mortality rates, much remains to be done to close the equity gap, particularly across provinces. Indeed, one of the main obstacles facing the ambitious government target of achieving universal health coverage by the year 2019 are the deficiencies in the availability and quality of health services for populations in remote and disadvantaged geographical areas. Concerted efforts are required to ensure that traditionally disadvantaged regions, such as NTMP, start catching-up. At a national level, there has been an emphasis on the mechanism to provide universal financial access to health through social insurance. In disadvantaged regions, universal health coverage will be contingent on substantial health system investments to ensure the necessary services are accessible to all.

## Supporting Information

File S1 Combined Supporting Information file containing: Table S1, Inequalities in under-five and neonatal mortality (per 1,000 live births) by wealth and education for all years, with 95% confidence intervals and p-values for trend. Table S2, Inequalities in under-five and neonatal mortality (per 1,000 live births) by rural/urban location and island division for all years, with 95% confidence intervals and p-values for trend. Table S3, Under-five mortality rates per 1,000 live births by equity marker. Table S4, Neonatal mortality rates per 1,000 live births by equity marker.
(DOCX)
