# Supplementary material for: Location Matters: Trends in Inequalities in Child Mortality in Indonesia. Evidence from Repeated Cross-Sectional Surveys
Source: PLoS One. 2014 Jul 25;9(7):e103597. doi: 10.1371/journal.pone.0103597 (PMC4111602; doi:10.1371/journal.pone.0103597)
Supplement: File S1 — Combined Supporting Information file containing: Table S1, Inequalities in under-five and neonatal mortality (per 1,000 live births) by wealth and education for all years, with 95% confidence intervals and p-values for trend. Table S2, Inequalities in under-five and neonatal mortality (per 1,000 live births) by rural/urban location and island division for all years, with 95% confidence intervals and p-values for trend. Table S3, Under-five mortality rates per 1,000 live births by equity marker. Table S4, Neonatal mortality rates per 1,000 live births by equity marker. (DOCX) [file pone.0103597.s001.docx]

**FILE S1: SUPPORTING INFORMATION**

**Table S1:** Inequalities in under-five and neonatal mortality (per 1,000 live births) by wealth and education for all years, with 95% confidence intervals and *p*-values for trend

| **Equity Marker** |  | **Relative Inequalities** | | | |  | **Absolute Inequalities** | | | |
| --- | --- | --- | --- | --- | --- | --- | --- | --- | --- | --- |
|  |  | **RR** | **95% CI** | **RII** | **95% CI** |  | **RD** | **95% CI** | **SII** | **95% CI** |
| **Wealth** |  |  |  |  |  |  |  |  |  |  |
| *U5MR* |  |  |  |  |  |  |  |  |  |  |
| 1980-81 |  | 2.03 | (1.73; 2.36) | 2.19 | (1.12; 3.25) |  | 74.3 | (59; 89.4) | 86.95 | (39.1; 134.8) |
| 1982-83 |  | 2.36 | (1.96; 2.81) | 2.17 | (0.07; 4.28) |  | 76.1 | (61.5; 89.2) | 80.78 | (-8.59; 170.16) |
| 1984-85 |  | 2.24 | (1.85; 2.71) | 2.40 | (0.73; 4.06) |  | 74.1 | (59.3; 88.9) | 86.81 | (27.5; 146.11) |
| 1986-87 |  | 2.85 | (2.36; 3.41) | 2.90 | (-0.09; 5.89) |  | 82.5 | (70.1; 95.1) | 96.61 | (21.6; 171.62) |
| 1988-89 |  | 2.84 | (2.25; 3.54) | 3.92 | (3.11; 4.74) |  | 89.0 | (71.7; 107.8) | 114.08 | (101.87; 126.3) |
| 1990-91 |  | 3.52 | (2.8; 4.43) | 5.68 | (3.54; 7.82) |  | 91.1 | (78.3; 104.4) | 117.59 | (102.62; 132.56) |
| 1992-93 |  | 3.96 | (3.06; 5.17) | 5.31 | (-0.27; 10.89) |  | 83.8 | (71.6; 98) | 100.50 | (62.06; 138.93) |
| 1994-95 |  | 3.12 | (2.28; 4.05) | 3.42 | (-0.04; 6.89) |  | 67.0 | (50.9; 82) | 76.52 | (29.24; 123.79) |
| 1996-97 |  | 3.91 | (2.83; 5.33) | 3.71 | (-1.74; 9.17) |  | 67.9 | (55.1; 79.9) | 72.39 | (13.72; 131.06) |
| 1998-99 |  | 3.67 | (2.52; 5.49) | 5.90 | (-0.21; 12.01) |  | 61.8 | (48.4; 74.2) | 77.85 | (51.7; 104) |
| 2000-01 |  | 3.07 | (2.18; 4.16) | 3.98 | (0.44; 7.52) |  | 55.7 | (41.4; 69) | 67.03 | (36.68; 97.37) |
| 2002-03 |  | 3.00 | (2.07; 4.18) | 4.15 | (-2.3; 10.6) |  | 53.4 | (38.4; 67.3) | 69.75 | (17.35; 122.16) |
| 2004-05 |  | 2.75 | (1.83; 4.23) | 3.40 | (-1.48; 8.28) |  | 45.3 | (29.5; 60.5) | 52.39 | (6.01; 98.77) |
| 2006-07 |  | 4.12 | (2.32; 6.37) | 7.61 | (-5.58; 20.81) |  | 46.1 | (30.5; 59.7) | 59.44 | (34.16; 84.71) |
| 2008-09 |  | 1.92 | (1.16; 3.3) | 2.96 | (0.03; 5.89) |  | 30.5 | (8; 51) | 44.18 | (12.13; 76.22) |
| 2010-11 |  | 2.33 | (1.24; 3.99) | 3.92 | (-5.58; 13.41) |  | 29.2 | (8.4; 46.1) | 40.26 | (-10.32; 90.85) |
| Trend [*p*-value] |  | 1.008 | [0.641] | 1.039 | [0.061] |  | -3.531 | [0.000] | -3.692 | [0.003] |
|  |  |  |  |  |  |  |  |  |  |  |
| *NMR* |  |  |  |  |  |  |  |  |  |  |
| 1980-81 |  | 1.57 | (1.21; 2.05) | 1.72 | (0.99; 2.44) |  | 17.9 | (8; 27.9) | 21.37 | (5.57; 37.16) |
| 1982-83 |  | 2.04 | (1.53; 2.76) | 1.95 | (-0.08; 3.98) |  | 19.6 | (12.1; 27.4) | 22.22 | (-9.43; 53.87) |
| 1984-85 |  | 1.86 | (1.3; 2.71) | 1.57 | (-0.06; 3.2) |  | 16.4 | (7.7; 24.5) | 14.38 | (-17.38; 46.15) |
| 1986-87 |  | 2.20 | (1.65; 3.08) | 2.32 | (0.1; 4.54) |  | 21.7 | (14.1; 30.1) | 26.84 | (0.33; 53.34) |
| 1988-89 |  | 1.82 | (1.16; 2.75) | 2.71 | (0.23; 5.2) |  | 21.2 | (5.1; 38) | 32.06 | (7.87; 56.26) |
| 1990-91 |  | 2.22 | (1.56; 3.28) | 2.71 | (1.55; 3.87) |  | 21.9 | (12.9; 31.4) | 26.88 | (17.4; 36.36) |
| 1992-93 |  | 2.45 | (1.69; 3.76) | 3.14 | (1.32; 4.96) |  | 25.0 | (15; 35.4) | 30.87 | (18.69; 43.06) |
| 1994-95 |  | 2.05 | (1.39; 3.15) | 1.53 | (-0.91; 3.97) |  | 17.3 | (8.6; 26.2) | 12.08 | (-31.72; 55.87) |
| 1996-97 |  | 2.32 | (1.55; 3.64) | 1.79 | (-0.98; 4.56) |  | 17.1 | (9.3; 25.2) | 14.15 | (-21.03; 49.33) |
| 1998-99 |  | 2.14 | (1.24; 4.32) | 2.38 | (0.39; 4.37) |  | 16.1 | (5.4; 25.2) | 18.76 | (3.14; 34.38) |
| 2000-01 |  | 2.27 | (1.42; 3.65) | 2.56 | (-2.03; 7.14) |  | 19.6 | (8.4; 30) | 21.22 | (-12.88; 55.31) |
| 2002-03 |  | 1.92 | (1.14; 3.53) | 2.65 | (-1.93; 7.23) |  | 12.5 | (2.9; 22.3) | 20.17 | (-9.65; 49.99) |
| 2004-05 |  | 2.46 | (1.32; 4.63) | 2.22 | (-0.64; 5.07) |  | 18.2 | (6.3; 28.5) | 17.49 | (-7.49; 42.47) |
| 2006-07 |  | 3.90 | (1.81; 9.87) | 4.41 | (-7.28; 16.11) |  | 18.0 | (8.1; 28) | 22.37 | (-4.31; 49.06) |
| 2008-09 |  | 2.01 | (0.95; 6.2) | 2.51 | (0.25; 4.76) |  | 15.0 | (-1.1; 29.7) | 19.43 | (3.32; 35.54) |
| 2010-11 |  | 2.01 | (0.86; 4.76) | 3.19 | (-1.7; 8.07) |  | 11.3 | (-2.6; 23.5) | 16.12 | (-0.38; 32.62) |
| Trend [*p*-value] |  | 1.019 | [0.111] | 1.033 | [0.001] |  | -0.398 | [0.031] | -0.378 | [0.142] |
|  |  |  |  |  |  |  |  |  |  |  |
| **Education** |  |  |  |  |  |  |  |  |  |  |
| *U5MR* |  |  |  |  |  |  |  |  |  |  |
| 1980-81 |  | 2.47 | (2.06; 2.91) | 2.67 | (0.32; 5.01) |  | 90.4 | (74.3; 106.4) | 103.75 | (26.7; 180.81) |
| 1982-83 |  | 2.94 | (2.46; 3.5) | 3.54 | (-1.2; 8.28) |  | 94.9 | (79.6; 108.9) | 119.76 | (25.53; 214) |
| 1984-85 |  | 2.89 | (2.42; 3.43) | 3.22 | (-0.55; 6.99) |  | 93.6 | (78; 107.2) | 107.87 | (24.5; 191.24) |
| 1986-87 |  | 2.71 | (2.28; 3.15) | 3.54 | (-1.92; 9) |  | 79.9 | (66.5; 94.5) | 107.44 | (10.09; 204.79) |
| 1988-89 |  | 2.47 | (1.98; 3.02) | 3.21 | (0.67; 5.76) |  | 75.4 | (57; 92.9) | 96.33 | (45.8; 146.86) |
| 1990-91 |  | 2.82 | (2.3; 3.43) | 4.11 | (-2.52; 10.75) |  | 88.5 | (70.1; 109.4) | 98.02 | (20.54; 175.49) |
| 1992-93 |  | 3.01 | (2.43; 3.77) | 4.44 | (-3.38; 12.26) |  | 79.7 | (61.5; 101.8) | 88.73 | (18.66; 158.8) |
| 1994-95 |  | 2.39 | (1.86; 3.04) | 3.85 | (2.9; 4.79) |  | 58.3 | (40.3; 80.9) | 76.47 | (66.51; 86.43) |
| 1996-97 |  | 1.82 | (1.11; 3.14) | 2.50 | (2.06; 2.94) |  | 38.6 | (7.5; 67.7) | 53.34 | (44.6; 62.07) |
| 1998-99 |  | 2.48 | (1.85; 3.33) | 4.47 | (-5.32; 14.25) |  | 54.5 | (35.7; 79.6) | 66.72 | (1.6; 131.83) |
| 2000-01 |  | 2.14 | (1.46; 3.01) | 3.78 | (-6.01; 13.57) |  | 46.6 | (21.6; 78.2) | 63.38 | (-25.85; 152.61) |
| 2002-03 |  | 3.50 | (2.41; 5.05) | 12.58 | (-31.15; 56.31) |  | 81.8 | (49.5; 122.3) | 93.54 | (46.12; 140.97) |
| 2004-05 |  | 1.84 | (1.35; 2.97) | 2.64 | (-1.21; 6.49) |  | 29.7 | (13.1; 65.5) | 41.10 | (-10.44; 92.64) |
| 2006-07 |  | 2.77 | (1.78; 4.82) | 6.03 | (-4.27; 16.32) |  | 45.5 | (22.1; 95.1) | 52.67 | (23.85; 81.5) |
| 2008-09 |  | 3.15 | (2.01; 5.25) | 8.49 | (-27.3; 44.29) |  | 67.2 | (36.5; 129.6) | 68.58 | (4.4; 132.75) |
| 2010-11 |  | 3.36 | (2.27; 6.5) | 5.47 | (-31.66; 42.61) |  | 62.1 | (37.6; 135) | 45.43 | (-65.06; 155.92) |
| Trend [*p*-value] |  | 1.003 | [0.783] | 1.053 | [0.001] |  | -2.977 | [0.002] | -4.450 | [0.000] |
|  |  |  |  |  |  |  |  |  |  |  |
| *NMR* |  |  |  |  |  |  |  |  |  |  |
| 1980-81 |  | 1.78 | (1.37; 2.32) | 1.87 | (0.87; 2.88) |  | 21.5 | (12; 31) | 24.21 | (5.02; 43.41) |
| 1982-83 |  | 2.11 | (1.56; 2.94) | 1.98 | (-1.6; 5.57) |  | 20.1 | (11.9; 29.3) | 22.55 | (-31.66; 76.76) |
| 1984-85 |  | 2.00 | (1.47; 2.69) | 2.13 | (0.49; 3.78) |  | 20.4 | (11.6; 29.1) | 23.29 | (2.11; 44.46) |
| 1986-87 |  | 2.07 | (1.54; 2.72) | 2.60 | (-0.44; 5.64) |  | 20.9 | (12.2; 29.6) | 29.22 | (-0.74; 59.19) |
| 1988-89 |  | 1.68 | (1.21; 2.36) | 1.75 | (-1.22; 4.72) |  | 14.2 | (4.6; 25) | 17.53 | (-32.41; 67.47) |
| 1990-91 |  | 2.29 | (1.55; 3.27) | 2.78 | (-3.15; 8.7) |  | 27.8 | (14.1; 42.1) | 27.30 | (-19.27; 73.88) |
| 1992-93 |  | 2.29 | (1.61; 3.3) | 2.77 | (-0.68; 6.22) |  | 26.1 | (13.6; 41.6) | 27.50 | (-0.08; 55.09) |
| 1994-95 |  | 1.81 | (1.1; 2.73) | 2.07 | (0.7; 3.44) |  | 17.2 | (2.5; 33.5) | 19.17 | (3.5; 34.84) |
| 1996-97 |  | 1.07 | (0.51; 2.98) | 1.06 | (-0.46; 2.57) |  | 2.0 | (-23.8; 24.6) | 1.54 | (-37.59; 40.66) |
| 1998-99 |  | 1.61 | (0.9; 2.67) | 1.90 | (1.07; 2.73) |  | 11.5 | (-2.3; 26.3) | 13.98 | (5.24; 22.71) |
| 2000-01 |  | 2.38 | (1.16; 4.04) | 3.09 | (-7.96; 14.14) |  | 27.3 | (3.9; 54.5) | 24.70 | (-36.82; 86.22) |
| 2002-03 |  | 2.17 | (0.8; 4) | 3.46 | (-2.36; 9.29) |  | 19.9 | (-3.9; 45.1) | 24.53 | (-0.41; 49.47) |
| 2004-05 |  | 1.84 | (0.88; 3.5) | 2.38 | (-1.82; 6.57) |  | 14.8 | (-2.3; 38.6) | 18.26 | (-13.97; 50.49) |
| 2006-07 |  | 0.83 | (0.27; 2.77) | 5.41 | (-27.48; 38.31) |  | -2.0 | (-10.4; 18.5) | 23.85 | (-28.43; 76.13) |
| 2008-09 |  | 1.80 | (0.64; 4.01) | 2.90 | (-2.28; 8.08) |  | 14.6 | (-6.8; 52.4) | 21.47 | (-7.71; 50.64) |
| 2010-11 |  | 1.10 | (0.43; 3.74) | 3.50 | (-15.18; 22.19) |  | 1.2 | (-8.5; 29.5) | 16.66 | (-36.69; 70.02) |
| Trend [*p*-value] |  | 0.971 | [0.023] | 1.041 | [0.001] |  | -1.028 | [0.004] | -0.356 | [0.055] |

*Notes*: U5MR, under-five mortality rate; NMR, neonatal mortality rate; CI, confidence interval; RR, rate ratio; RD, rate difference; RII, relative index of inequality; SII, slope index of inequality. The small number of observations and possible non-linear relationships implies that the trend estimates should be treated with caution.

**Table S2:** Inequalities in under-five and neonatal mortality (per 1,000 live births) by rural/urban location and island division for all years, with 95% confidence intervals and *p*-values for trend

| **Equity Marker** |  | | | **U5MR** | | | | |  | **NMR** | | | |
| --- | --- | --- | --- | --- | --- | --- | --- | --- | --- | --- | --- | --- | --- |
|  |  | | | **RR** | | **95% CI** | **RD** | **95% CI** |  | **RR** | **95% CI** | **RD** | **95% CI** |
| **Urban/Rural (base = Urban)** | | | | | | |  |  |  |  |  |  |  |
| *Rural* |  |  | | |  | |  |  |  |  |  |  |  |
| 1980-81 |  | 1.24 | | | (1.12; 1.39) | | 24.3 | (13.28; 35.71) |  | 1.41 | (1.18; 1.7) | 13.0 | (6.25; 19.55) |
| 1982-83 |  | 1.35 | | | (1.22; 1.51) | | 31.2 | (21.07; 41.29) |  | 1.34 | (1.11; 1.64) | 9.6 | (3.4; 15.36) |
| 1984-85 |  | 1.37 | | | (1.2; 1.53) | | 30.8 | (18.51; 41.13) |  | 1.30 | (1.04; 1.63) | 8.1 | (1.43; 14.31) |
| 1986-87 |  | 1.63 | | | (1.46; 1.83) | | 43.1 | (33.53; 51.79) |  | 1.56 | (1.26; 1.94) | 13.4 | (7.24; 19.27) |
| 1988-89 |  | 1.47 | | | (1.28; 1.67) | | 33.4 | (21.77; 44.35) |  | 1.35 | (1.03; 1.76) | 9.5 | (1; 17.41) |
| 1990-91 |  | 1.75 | | | (1.52; 2) | | 40.3 | (31.36; 48.9) |  | 1.90 | (1.51; 2.41) | 16.0 | (10.64; 21.36) |
| 1992-93 |  | 1.57 | | | (1.33; 1.84) | | 29.2 | (19.13; 38.17) |  | 1.63 | (1.25; 2.1) | 12.8 | (6.16; 18.76) |
| 1994-95 |  | 1.39 | | | (1.02; 1.91) | | 21.6 | (1.47; 38.23) |  | 1.08 | (0.67; 1.85) | 2.3 | (-13.51; 15.32) |
| 1996-97 |  | 1.38 | | | (1.01; 1.89) | | 19.4 | (0.59; 35.05) |  | 1.00 | (0.64; 1.76) | 0.0 | (-13.28; 12.25) |
| 1998-99 |  | 1.72 | | | (1.33; 2.23) | | 27.0 | (15.07; 36.99) |  | 1.22 | (0.81; 1.96) | 4.5 | (-5.24; 13.05) |
| 2000-01 |  | 1.43 | | | (1.14; 1.81) | | 18.7 | (7.28; 30.11) |  | 1.18 | (0.88; 1.59) | 3.7 | (-2.91; 10.41) |
| 2002-03 |  | 1.83 | | | (1.42; 2.36) | | 29.8 | (17.85; 43.47) |  | 1.72 | (1.2; 2.55) | 10.7 | (3.74; 18.13) |
| 2004-05 |  | 1.01 | | | (0.66; 1.7) | | 0.5 | (-25.81; 21.25) |  | 0.71 | (0.39; 1.84) | -9.3 | (-34.7; 10.43) |
| 2006-07 |  | 1.95 | | | (1.4; 2.72) | | 22.5 | (11.65; 32.51) |  | 2.18 | (1.32; 4.2) | 12.3 | (4.38; 20.46) |
| 2008-09 |  | 1.50 | | | (1.05; 2.07) | | 16.8 | (1.96; 29.81) |  | 1.42 | (0.88; 2.59) | 7.5 | (-2.82; 18.67) |
| 2010-11 |  | 1.66 | | | (1.13; 2.22) | | 15.5 | (3.89; 25.09) |  | 1.45 | (0.86; 2.55) | 5.3 | (-2.16; 13.4) |
| Trend [*p*-value] |  | 1.009 | | | [0.151] | | -1.377 | [0.005] |  | 0.997 | [0.784] | -0.560 | [0.030] |
|  |  |  | | |  | |  |  |  |  |  |  |  |
| **Island Division (base = Java and Bali)** | | | | | | |  |  |  |  |  |  |  |
| *Sumatra* |  | |  | |  | |  |  |  |  |  |  |  |
| 1980-81 |  | | 0.99 | | (0.9; 1.09) | | -0.9 | (-11.7; 9.83) |  | 0.96 | (0.81; 1.15) | -1.4 | (-8.26; 5.29) |
| 1982-83 |  | | 0.92 | | (0.83; 1.01) | | -9.1 | (-19.71; 1.51) |  | 0.92 | (0.77; 1.11) | -2.8 | (-8.64; 3.48) |
| 1984-85 |  | | 0.85 | | (0.76; 0.95) | | -15.8 | (-26.46; -4.84) |  | 0.76 | (0.62; 0.93) | -8.5 | (-14.73; -2.19) |
| 1986-87 |  | | 0.93 | | (0.83; 1.04) | | -6.7 | (-18.05; 3.69) |  | 0.85 | (0.7; 1.04) | -5.2 | (-11.69; 1.37) |
| 1988-89 |  | | 0.88 | | (0.77; 0.99) | | -11.4 | (-23.06; -0.98) |  | 0.69 | (0.54; 0.89) | -11.6 | (-20; -3.32) |
| 1990-91 |  | | 1.15 | | (1.01; 1.31) | | 11.2 | (0.44; 21.46) |  | 1.06 | (0.85; 1.32) | 1.6 | (-5.09; 7.92) |
| 1992-93 |  | | 1.33 | | (1.13; 1.54) | | 19.8 | (8.9; 30.76) |  | 1.03 | (0.78; 1.31) | 0.8 | (-6.88; 7.69) |
| 1994-95 |  | | 1.04 | | (0.86; 1.24) | | 2.8 | (-10.27; 13.75) |  | 0.85 | (0.61; 1.17) | -4.4 | (-14.3; 4.16) |
| 1996-97 |  | | 1.27 | | (1.02; 1.59) | | 14.3 | (1.39; 29.17) |  | 1.36 | (0.91; 2.16) | 8.0 | (-2.58; 20.51) |
| 1998-99 |  | | 1.22 | | (0.96; 1.49) | | 9.5 | (-1.94; 18.44) |  | 1.07 | (0.75; 1.52) | 1.5 | (-6.49; 8.64) |
| 2000-01 |  | | 1.27 | | (1.02; 1.55) | | 12.0 | (1.07; 22.22) |  | 1.31 | (0.98; 1.87) | 6.4 | (-0.53; 14.24) |
| 2002-03 |  | | 0.98 | | (0.73; 1.34) | | -1.0 | (-17.04; 14.44) |  | 1.13 | (0.71; 1.8) | 2.5 | (-6.76; 12.46) |
| 2004-05 |  | | 1.53 | | (1.08; 2.1) | | 17.9 | (3.28; 30.53) |  | 1.59 | (0.92; 2.78) | 10.2 | (-2.02; 19.75) |
| 2006-07 |  | | 1.37 | | (0.96; 1.98) | | 10.2 | (-1.42; 23.12) |  | 0.95 | (0.59; 1.61) | -0.8 | (-8.99; 6.68) |
| 2008-09 |  | | 0.97 | | (0.65; 1.39) | | -1.3 | (-17.48; 13.21) |  | 1.28 | (0.77; 2.34) | 5.5 | (-6.29; 16.79) |
| 2010-11 |  | | 0.98 | | (0.7; 1.57) | | -0.5 | (-10.55; 13.02) |  | 1.14 | (0.63; 2.13) | 2.0 | (-6.65; 10.79) |
| Trend [*p*-value] |  | | 1.017 | | [0.133] | | 0.991 | [0.114] |  | 1.030 | [0.002] | 0.764 | [0.004] |
|  |  | |  | |  | |  |  |  |  |  |  |  |
| *Kalimantan, Sulawesi & NTMP* | | | | | | |  |  |  |  |  |  |  |
| 1980-81 |  | | 1.18 | | (1.07; 1.29) | | 19.8 | (7.72; 31.85) |  | 1.07 | (0.91; 1.26) | 2.9 | (-4.14; 9.54) |
| 1982-83 |  | | 1.17 | | (1.06; 1.29) | | 18.4 | (6.63; 29.79) |  | 1.13 | (0.95; 1.35) | 4.6 | (-1.82; 10.94) |
| 1984-85 |  | | 1.14 | | (1.02; 1.25) | | 14.4 | (2.56; 25.83) |  | 0.91 | (0.74; 1.11) | -3.1 | (-9.91; 3.77) |
| 1986-87 |  | | 1.15 | | (1.03; 1.29) | | 14.8 | (2.64; 25.86) |  | 0.96 | (0.79; 1.18) | -1.5 | (-8.06; 5.48) |
| 1988-89 |  | | 1.21 | | (1.07; 1.37) | | 19.5 | (6.94; 31.48) |  | 0.91 | (0.73; 1.16) | -3.3 | (-12; 5.02) |
| 1990-91 |  | | 1.36 | | (1.2; 1.54) | | 26.6 | (16.41; 36.91) |  | 1.06 | (0.86; 1.33) | 1.7 | (-4.4; 8.14) |
| 1992-93 |  | | 1.46 | | (1.25; 1.67) | | 27.6 | (16.95; 37.83) |  | 1.16 | (0.92; 1.45) | 4.4 | (-2.46; 10.75) |
| 1994-95 |  | | 1.24 | | (1.03; 1.46) | | 15.1 | (1.95; 26.57) |  | 0.87 | (0.64; 1.21) | -4.0 | (-13.16; 4.86) |
| 1996-97 |  | | 1.35 | | (1.13; 1.68) | | 18.9 | (7.96; 31.01) |  | 1.16 | (0.84; 1.71) | 3.5 | (-4.61; 11.48) |
| 1998-99 |  | | 1.71 | | (1.4; 2.09) | | 30.8 | (20.13; 41.29) |  | 1.28 | (0.92; 1.81) | 5.7 | (-2.16; 13.23) |
| 2000-01 |  | | 1.62 | | (1.28; 2.02) | | 27.5 | (14.76; 41.5) |  | 1.21 | (0.85; 1.77) | 4.5 | (-3.76; 13.04) |
| 2002-03 |  | | 1.26 | | (0.99; 1.69) | | 13.6 | (-0.78; 27.83) |  | 1.40 | (1.01; 2.07) | 7.7 | (0.14; 15.82) |
| 2004-05 |  | | 1.95 | | (1.38; 2.62) | | 32.2 | (17.07; 44.7) |  | 1.69 | (1.01; 2.92) | 11.8 | (0.27; 21.86) |
| 2006-07 |  | | 1.93 | | (1.41; 2.66) | | 25.7 | (14.31; 37.85) |  | 1.26 | (0.78; 2.1) | 4.2 | (-4.87; 12.29) |
| 2008-09 |  | | 1.29 | | (0.87; 1.77) | | 11.4 | (-6.76; 25.54) |  | 1.19 | (0.69; 2.25) | 3.7 | (-7.68; 15.04) |
| 2010-11 |  | | 1.63 | | (1.15; 2.36) | | 17.5 | (4.76; 31.06) |  | 1.07 | (0.58; 2.02) | 1.0 | (-7.5; 9.61) |
| Trend [*p*-value] |  | | 1.027 | | [0.002] | | 0.192 | [0.608] |  | 1.020 | [0.052] | 0.384 | [0.137] |

*Notes*: U5MR, under-five mortality rate; NMR, neonatal mortality rate; CI, confidence interval; RR, rate ratio; RD, rate difference. The small number of observations and possible non-linear relationships implies that the trend estimates should be treated with caution.

**Table S3:** Under-five mortality rates per 1,000 live births by equity marker

| **Equity Marker** | **U5MR** | **95% CI** | **U5MR** | **95% CI** | **U5MR** | **95% CI** |
| --- | --- | --- | --- | --- | --- | --- |
| **National** |  |  |  |  |  |  |
| 1980-81 | 116.3 | (111; 121.9) |  |  |  |  |
| 1982-83 | 109.5 | (104.6; 115) |  |  |  |  |
| 1984-85 | 105.0 | (100; 110.8) |  |  |  |  |
| 1986-87 | 98.3 | (93.2; 103.8) |  |  |  |  |
| 1988-89 | 93.9 | (88; 100.2) |  |  |  |  |
| 1990-91 | 80.7 | (76.4; 85.7) |  |  |  |  |
| 1992-93 | 70.5 | (65.9; 75.7) |  |  |  |  |
| 1994-95 | 67.2 | (61.7; 74.1) |  |  |  |  |
| 1996-97 | 60.5 | (54.8; 66.8) |  |  |  |  |
| 1998-99 | 51.9 | (47.9; 57.4) |  |  |  |  |
| 2000-01 | 53.5 | (49; 59.2) |  |  |  |  |
| 2002-03 | 54.3 | (47.5; 63.1) |  |  |  |  |
| 2004-05 | 45.9 | (40; 52.7) |  |  |  |  |
| 2006-07 | 36.1 | (31.8; 42.2) |  |  |  |  |
| 2008-09 | 42.1 | (35.9; 49.5) |  |  |  |  |
| 2010-11 | 31.5 | (27; 38) |  |  |  |  |
| Trend [*p*-value] | -5.67 | [0.000] |  |  |  |  |
|  |  |  |  |  |  |  |
| **Urban/Rural** | Urban |  | Rural |  |  |  |
| 1980-81 | 99.7 | (91; 109.3) | 124.0 | (117.6; 131) |  |  |
| 1982-83 | 88.1 | (80.6; 96.7) | 119.3 | (113.5; 125.8) |  |  |
| 1984-85 | 83.9 | (76; 94.3) | 114.7 | (108.3; 121.5) |  |  |
| 1986-87 | 68.5 | (61.9; 75.7) | 111.5 | (105.1; 118.2) |  |  |
| 1988-89 | 71.4 | (64.1; 80.9) | 104.8 | (98.1; 112.8) |  |  |
| 1990-91 | 53.8 | (47.7; 61) | 94.1 | (88.5; 100.3) |  |  |
| 1992-93 | 50.9 | (44.3; 59) | 80.1 | (74.7; 86.5) |  |  |
| 1994-95 | 55.7 | (41; 74.1) | 77.4 | (70; 85.8) |  |  |
| 1996-97 | 50.8 | (38; 68.3) | 70.2 | (62.8; 78.2) |  |  |
| 1998-99 | 37.4 | (29.2; 47.8) | 64.3 | (58.7; 71.4) |  |  |
| 2000-01 | 43.9 | (35.7; 53.7) | 62.5 | (56.1; 70.3) |  |  |
| 2002-03 | 36.1 | (30; 44.8) | 66.0 | (57.5; 77.8) |  |  |
| 2004-05 | 49.8 | (30.5; 75.8) | 50.3 | (44; 57.4) |  |  |
| 2006-07 | 23.7 | (17.9; 31.5) | 46.2 | (39.3; 54.8) |  |  |
| 2008-09 | 33.6 | (26.1; 46.8) | 50.5 | (42.7; 61.4) |  |  |
| 2010-11 | 23.6 | (18.4; 32.3) | 39.1 | (31.9; 48.5) |  |  |
| Trend [*p*-value] | -4.45 | [0.000] | -5.82 | [0.000] |  |  |
|  |  |  |  |  |  |  |
| **Wealth** | Lowest Quin. | | 2nd Quin. | | Middle Quin. | |
| 1980-81 | 146.4 | (135.2; 159.7) | 128.1 | (117.3; 141.9) | 124.8 | (113.8; 138.1) |
| 1982-83 | 132.1 | (122.1; 143.3) | 121.5 | (111.6; 133.9) | 119.1 | (108.5; 132.7) |
| 1984-85 | 133.6 | (124.2; 144.1) | 115.3 | (104.8; 126.9) | 118.4 | (107.7; 132.4) |
| 1986-87 | 127.0 | (117; 138.1) | 113.5 | (101.8; 126.5) | 113.5 | (100.6; 129.4) |
| 1988-89 | 137.3 | (123.5; 154.6) | 108.2 | (96.9; 120.5) | 91.4 | (79.4; 108.4) |
| 1990-91 | 127.2 | (116.3; 138.1) | 96.1 | (85.3; 109.7) | 69.9 | (61.2; 82) |
| 1992-93 | 112.2 | (102.9; 124.7) | 76.8 | (65.8; 89.4) | 61.9 | (53.9; 74.5) |
| 1994-95 | 98.5 | (87.1; 112) | 74.3 | (63.6; 88.1) | 57.1 | (47.3; 69.2) |
| 1996-97 | 91.2 | (82; 102) | 59.6 | (50.5; 71.2) | 59.9 | (50.3; 73.7) |
| 1998-99 | 85.0 | (75.8; 95) | 59.4 | (48.9; 73) | 41.9 | (33.6; 53.8) |
| 2000-01 | 82.7 | (72.5; 93.7) | 54.3 | (46.8; 66.6) | 51.9 | (40.4; 64.7) |
| 2002-03 | 80.2 | (71; 93.6) | 60.6 | (48.5; 77.7) | 68.0 | (44.7; 105.3) |
| 2004-05 | 71.3 | (61.5; 84.4) | 43.5 | (36.2; 60.2) | 37.1 | (27; 50.6) |
| 2006-07 | 60.8 | (49.7; 75.3) | 39.1 | (29.9; 53.6) | 39.7 | (28.6; 57.6) |
| 2008-09 | 63.8 | (50.5; 81) | 45.8 | (33.7; 64.6) | 34.1 | (25.8; 53.6) |
| 2010-11 | 51.1 | (39.5; 65.9) | 28.3 | (22.3; 44.6) | 40.9 | (29.5; 63.3) |
| Trend [*p*-value] | -6.37 | [0.000] | -6.70 | [0.000] | -6.22 | [0.000] |
|  |  |  |  |  |  |  |
|  | 4th Quin. | | Highest Quin. | |  |  |
| 1980-81 | 104.9 | (92.4; 118) | 72.1 | (63.5; 82.7) |  |  |
| 1982-83 | 110.1 | (99.5; 123.2) | 56.0 | (48.1; 66.5) |  |  |
| 1984-85 | 88.5 | (78.2; 101.2) | 59.6 | (49.8; 71.7) |  |  |
| 1986-87 | 80.2 | (70.4; 92.2) | 44.6 | (37.7; 53.2) |  |  |
| 1988-89 | 66.5 | (58; 77.6) | 48.4 | (39.8; 58.6) |  |  |
| 1990-91 | 56.9 | (48.9; 67.7) | 36.1 | (29.4; 45.4) |  |  |
| 1992-93 | 58.5 | (50; 71) | 28.4 | (22.3; 36) |  |  |
| 1994-95 | 65.3 | (46.5; 91.1) | 31.5 | (24.9; 41.9) |  |  |
| 1996-97 | 59.8 | (43.7; 87.4) | 23.3 | (17.6; 31.3) |  |  |
| 1998-99 | 40.5 | (30.4; 58.1) | 23.1 | (15.6; 33) |  |  |
| 2000-01 | 43.4 | (32.6; 61.6) | 27.0 | (20.5; 37.4) |  |  |
| 2002-03 | 30.6 | (23.2; 40.6) | 26.7 | (20.2; 38) |  |  |
| 2004-05 | 44.8 | (27.5; 70.1) | 25.9 | (17.5; 37.9) |  |  |
| 2006-07 | 20.5 | (15.4; 31.4) | 14.8 | (9.9; 25.3) |  |  |
| 2008-09 | 31.5 | (19.4; 53.7) | 33.3 | (20.3; 52.9) |  |  |
| 2010-11 | 14.3 | (9.9; 27.2) | 21.9 | (14.1; 38.2) |  |  |
| Trend [*p*-value] | -5.63 | [0.000] | -2.84 | [0.000] |  |  |
|  |  |  |  |  |  |  |
| **Education** | None | | Some Primary | | Complete Primary | |
| 1980-81 | 151.7 | (139.6; 166.8) | 126.5 | (119.2; 136.7) | 104.4 | (94.8; 115.3) |
| 1982-83 | 143.8 | (132; 157.5) | 130.5 | (122; 140.3) | 90.1 | (81.6; 99.7) |
| 1984-85 | 143.2 | (130.4; 157.1) | 117.1 | (108.8; 126.1) | 96.0 | (86.4; 106.7) |
| 1986-87 | 126.5 | (114; 140.4) | 121.9 | (112.4; 132.8) | 85.2 | (77.8; 94.8) |
| 1988-89 | 126.6 | (113.1; 143) | 111.2 | (102.8; 121.4) | 86.9 | (77.8; 98.8) |
| 1990-91 | 137.2 | (120.8; 157.2) | 93.4 | (86.1; 103.3) | 73.1 | (65.9; 81.8) |
| 1992-93 | 119.4 | (103.5; 140.3) | 83.9 | (75.2; 94.3) | 74.0 | (65.5; 84.4) |
| 1994-95 | 100.3 | (84; 122.7) | 87.8 | (75.9; 102.9) | 65.8 | (58.1; 75.6) |
| 1996-97 | 85.5 | (66.7; 108.7) | 80.2 | (69; 96) | 64.9 | (57.2; 75.7) |
| 1998-99 | 91.3 | (75.3; 116.4) | 79.9 | (68; 94) | 50.4 | (42.5; 61) |
| 2000-01 | 87.4 | (65.2; 118.4) | 87.7 | (74.7; 106) | 51.0 | (43.8; 59.9) |
| 2002-03 | 114.5 | (84.2; 154.6) | 84.4 | (69.4; 103.9) | 67.5 | (52; 90) |
| 2004-05 | 64.9 | (48.9; 100.3) | 53.7 | (41.9; 71.4) | 59.2 | (46.4; 76.2) |
| 2006-07 | 71.1 | (48; 121.6) | 53.2 | (40; 72.9) | 47.8 | (40.3; 64.1) |
| 2008-09 | 98.5 | (68.1; 159.3) | 72.5 | (43.3; 114.6) | 52.7 | (40.1; 72.3) |
| 2010-11 | 88.4 | (66.6; 162.8) | 59.4 | (39.3; 95.9) | 31.6 | (22.8; 45.4) |
| Trend [*p*-value] | -4.88 | [0.000] | -4.87 | [0.000] | -3.91 | [0.000] |
|  |  |  |  |  |  |  |
|  | Some secondary or higher | |  |  |  |  |
| 1980-81 | 61.3 | (53.4; 72.2) |  |  |  |  |
| 1982-83 | 48.9 | (42.1; 57.5) |  |  |  |  |
| 1984-85 | 49.6 | (42.7; 57.4) |  |  |  |  |
| 1986-87 | 46.6 | (41.6; 53.4) |  |  |  |  |
| 1988-89 | 51.2 | (43.6; 61.3) |  |  |  |  |
| 1990-91 | 48.7 | (42.4; 56.8) |  |  |  |  |
| 1992-93 | 39.7 | (34.6; 46.5) |  |  |  |  |
| 1994-95 | 42.0 | (35.8; 50.4) |  |  |  |  |
| 1996-97 | 46.9 | (30.2; 70.4) |  |  |  |  |
| 1998-99 | 36.8 | (31.1; 45.2) |  |  |  |  |
| 2000-01 | 40.8 | (35; 49.6) |  |  |  |  |
| 2002-03 | 32.7 | (27.3; 39.5) |  |  |  |  |
| 2004-05 | 35.2 | (29.4; 42.4) |  |  |  |  |
| 2006-07 | 25.6 | (21.3; 31.9) |  |  |  |  |
| 2008-09 | 31.2 | (25.3; 40.5) |  |  |  |  |
| 2010-11 | 26.3 | (21; 34.4) |  |  |  |  |
| Trend [*p*-value] | -1.90 | [0.000] |  |  |  |  |
|  |  |  |  |  |  |  |
| **Island** | Java & Bali | | Sumatra | | Kalimantan, Sulawesi & NTMP | |
| 1980-81 | 113.1 | (105.7; 121.2) | 112.2 | (104.7; 121.3) | 133.0 | (124.9; 142.2) |
| 1982-83 | 108.4 | (101.5; 117.5) | 99.3 | (92.7; 106.2) | 126.8 | (119.5; 135.3) |
| 1984-85 | 106.3 | (97.8; 114.9) | 90.5 | (84.1; 97.9) | 120.7 | (113.1; 129.1) |
| 1986-87 | 97.2 | (89.4; 106.6) | 90.5 | (83.5; 97.7) | 112.1 | (104.9; 120.2) |
| 1988-89 | 92.8 | (84; 103.3) | 81.4 | (75.6; 88.2) | 112.3 | (105.4; 120.2) |
| 1990-91 | 73.0 | (66.2; 81.7) | 84.2 | (77.5; 91.5) | 99.6 | (93.3; 107.4) |
| 1992-93 | 60.7 | (54.7; 69.5) | 80.4 | (73.3; 89) | 88.3 | (81.9; 96.3) |
| 1994-95 | 63.6 | (55.2; 74.6) | 66.4 | (58.8; 74) | 78.7 | (72.1; 87.1) |
| 1996-97 | 53.6 | (45.4; 64.3) | 68.0 | (59.7; 79.9) | 72.5 | (66.3; 80.5) |
| 1998-99 | 43.3 | (36.4; 52.4) | 52.8 | (46.2; 59.7) | 74.0 | (67.6; 83.2) |
| 2000-01 | 44.7 | (37.8; 53.5) | 56.8 | (50.5; 64.6) | 72.3 | (63.9; 83.5) |
| 2002-03 | 51.4 | (40; 65.9) | 50.4 | (42.4; 60.7) | 65.0 | (58.1; 74.7) |
| 2004-05 | 34.1 | (26.1; 46.6) | 52.0 | (43.9; 62.4) | 66.3 | (58.3; 77.8) |
| 2006-07 | 27.7 | (21.4; 37.2) | 37.9 | (31.2; 49.5) | 53.4 | (45.3; 64.5) |
| 2008-09 | 39.8 | (31.7; 54.9) | 38.6 | (31.5; 50.7) | 51.2 | (41.8; 63.8) |
| 2010-11 | 27.7 | (20.8; 38) | 27.2 | (22.2; 39.1) | 45.1 | (36.1; 58.6) |
| Trend [*p*-value] | -6.03 | [0.000] | -5.04 | [0.000] | -5.84 | [0.000] |

*Notes*: U5MR, under-five mortality rate; CI, confidence interval; RR, rate ratio; RD, rate difference; Quin., Quintile; NTMP, Nusa Tenggara, Maluku and Papua. The small number of observations and possible non-linear relationships implies that the trend estimates should be treated with caution.

**Table S4:** Neonatal mortality rates per 1,000 live births by equity marker

| **Equity Marker** | **NMR** | **95% CI** | **NMR** | **95% CI** | **NMR** | **95% CI** |
| --- | --- | --- | --- | --- | --- | --- |
| **National** |  |  |  |  |  |  |
| 1980-81 | 40.4 | (37.2; 43.7) |  |  |  |  |
| 1982-83 | 34.8 | (31.8; 37.7) |  |  |  |  |
| 1984-85 | 32.7 | (29.8; 36.1) |  |  |  |  |
| 1986-87 | 33.6 | (30.4; 36.8) |  |  |  |  |
| 1988-89 | 34.1 | (29.7; 38.7) |  |  |  |  |
| 1990-91 | 28.4 | (25.5; 31.4) |  |  |  |  |
| 1992-93 | 28.8 | (25.4; 32.2) |  |  |  |  |
| 1994-95 | 28.7 | (23.8; 34.2) |  |  |  |  |
| 1996-97 | 24.7 | (20.3; 29.6) |  |  |  |  |
| 1998-99 | 22.3 | (18.8; 26.4) |  |  |  |  |
| 2000-01 | 23.3 | (19.8; 26.7) |  |  |  |  |
| 2002-03 | 21.6 | (18.3; 25.7) |  |  |  |  |
| 2004-05 | 22.4 | (18; 27.8) |  |  |  |  |
| 2006-07 | 16.9 | (13; 21.2) |  |  |  |  |
| 2008-09 | 21.5 | (16.3; 27) |  |  |  |  |
| 2010-11 | 14.4 | (10.9; 18.7) |  |  |  |  |
| Trend [*p*-value] | -1.43 | [0.000] |  |  |  |  |
|  |  |  |  |  |  |  |
| **Urban/Rural** | Urban |  | Rural |  |  |  |
| 1980-81 | 31.4 | (26.8; 36.5) | 44.4 | (40.4; 48.4) |  |  |
| 1982-83 | 28.2 | (23.8; 32.9) | 37.8 | (34.1; 41.3) |  |  |
| 1984-85 | 27.0 | (21.9; 33) | 35.1 | (31.4; 39.2) |  |  |
| 1986-87 | 24.2 | (20; 28.8) | 37.6 | (33.6; 41.7) |  |  |
| 1988-89 | 27.5 | (21.8; 33.5) | 37.0 | (31.1; 42.7) |  |  |
| 1990-91 | 17.8 | (14.4; 22) | 33.7 | (30; 37.8) |  |  |
| 1992-93 | 20.4 | (16; 25.1) | 33.2 | (29; 37.7) |  |  |
| 1994-95 | 28.4 | (17.5; 43.2) | 30.8 | (25.4; 37.2) |  |  |
| 1996-97 | 26.0 | (14.7; 38.6) | 26.0 | (20.7; 31.2) |  |  |
| 1998-99 | 20.3 | (13.1; 29.3) | 24.7 | (20.5; 29.2) |  |  |
| 2000-01 | 21.1 | (16.9; 26.1) | 24.8 | (20.6; 29.8) |  |  |
| 2002-03 | 14.9 | (10.7; 20.2) | 25.6 | (21; 31.1) |  |  |
| 2004-05 | 31.8 | (13.3; 58.5) | 22.5 | (18.2; 27.2) |  |  |
| 2006-07 | 10.4 | (5.5; 15.6) | 22.7 | (17.2; 29.3) |  |  |
| 2008-09 | 17.7 | (10.5; 25.3) | 25.2 | (18.9; 33.7) |  |  |
| 2010-11 | 11.7 | (7.3; 16.7) | 17.0 | (11.5; 23.5) |  |  |
| Trend [*p*-value] | -0.91 | [0.000] | -1.47 | [0.000] |  |  |
|  |  |  |  |  |  |  |
| **Wealth** | Lowest Quin. | | 2nd Quin. |  | Middle Quin. | |
| 1980-81 | 49.5 | (42.1; 57.2) | 40.6 | (33.8; 47.7) | 43.9 | (36.5; 52.6) |
| 1982-83 | 38.3 | (33.1; 44.2) | 41.8 | (35.2; 48.9) | 37.7 | (31.1; 45.4) |
| 1984-85 | 35.5 | (29.9; 41) | 34.6 | (28; 41.5) | 34.8 | (28; 42.5) |
| 1986-87 | 39.8 | (33.5; 46.5) | 41.0 | (33.4; 49.9) | 37.8 | (30.2; 46.2) |
| 1988-89 | 46.9 | (33.4; 61.9) | 38.0 | (30; 46.4) | 35.2 | (27.6; 44.2) |
| 1990-91 | 39.9 | (33.1; 47.3) | 29.7 | (23.1; 37.6) | 26.3 | (19.8; 34.2) |
| 1992-93 | 42.3 | (34.6; 50.6) | 29.7 | (22.4; 37.4) | 26.9 | (20.1; 34.2) |
| 1994-95 | 33.8 | (27.9; 40.8) | 27.7 | (21.6; 35.9) | 23.8 | (16.7; 31.3) |
| 1996-97 | 30.1 | (23.8; 37) | 25.5 | (18.6; 32.8) | 20.2 | (15.1; 26.8) |
| 1998-99 | 30.1 | (24.9; 35.8) | 24.9 | (16; 34.4) | 17.6 | (12.9; 23.7) |
| 2000-01 | 35.0 | (26.6; 43.6) | 16.5 | (12.2; 21.7) | 27.0 | (18.3; 36.3) |
| 2002-03 | 26.2 | (19.9; 34.2) | 33.4 | (23.3; 48.4) | 18.4 | (10.5; 27.6) |
| 2004-05 | 30.7 | (22.5; 39.5) | 19.2 | (12.6; 27.5) | 23.4 | (14; 34.8) |
| 2006-07 | 24.3 | (16.1; 33.9) | 18.8 | (9.9; 28.6) | 24.5 | (12.1; 38.7) |
| 2008-09 | 30.0 | (20.1; 41.4) | 21.6 | (11.9; 34) | 25.1 | (15.5; 39.7) |
| 2010-11 | 22.5 | (14.1; 33.1) | 13.8 | (7.6; 23.6) | 16.3 | (8; 27.9) |
| Trend [*p*-value] | -1.37 | [0.000] | -1.69 | [0.000] | -1.42 | [0.000] |
|  |  |  |  |  |  |  |
|  | 4th Quin. |  | Highest Quin. | |  |  |
| 1980-81 | 34.9 | (28; 41.9) | 31.6 | (25.9; 38.5) |  |  |
| 1982-83 | 34.2 | (27.3; 42.4) | 18.8 | (14.5; 24.3) |  |  |
| 1984-85 | 36.9 | (28.9; 45) | 19.1 | (13.6; 25.8) |  |  |
| 1986-87 | 27.5 | (21.2; 34) | 18.1 | (13.5; 23.4) |  |  |
| 1988-89 | 19.4 | (13.9; 25.2) | 25.7 | (18.7; 33.1) |  |  |
| 1990-91 | 24.1 | (18.3; 30.3) | 18.0 | (13; 24.2) |  |  |
| 1992-93 | 23.6 | (17.4; 31.7) | 17.3 | (11.7; 23.2) |  |  |
| 1994-95 | 39.9 | (18.3; 63.9) | 16.5 | (11.2; 22.7) |  |  |
| 1996-97 | 32.7 | (18.2; 58.2) | 13.0 | (8.2; 17.9) |  |  |
| 1998-99 | 22.5 | (12.1; 37.8) | 14.0 | (7.8; 23.3) |  |  |
| 2000-01 | 20.4 | (12.7; 30.1) | 15.4 | (9.9; 22.7) |  |  |
| 2002-03 | 14.9 | (9.9; 22.8) | 13.7 | (7.3; 21.6) |  |  |
| 2004-05 | 24.4 | (8.5; 46.5) | 12.5 | (7; 19.4) |  |  |
| 2006-07 | 8.6 | (4.5; 15.6) | 6.2 | (2.2; 11.8) |  |  |
| 2008-09 | 15.1 | (2.9; 28.6) | 14.9 | (5.3; 27.6) |  |  |
| 2010-11 | 8.1 | (3; 14.9) | 11.2 | (5.2; 21.1) |  |  |
| Trend [*p*-value] | -1.54 | [0.000] | -0.97 | [0.001] |  |  |
|  |  |  |  |  |  |  |
| **Education** | None |  | Some Primary | | Complete Primary | |
| 1980-81 | 49.1 | (42; 57.3) | 42.4 | (37.3; 48.1) | 38.0 | (32.1; 44.2) |
| 1982-83 | 38.2 | (30.9; 46) | 39.5 | (34.3; 44.6) | 35.7 | (30; 41.6) |
| 1984-85 | 40.8 | (33.7; 48.2) | 35.4 | (30.4; 41.2) | 31.2 | (25.1; 37.5) |
| 1986-87 | 40.5 | (33.1; 48.5) | 40.6 | (34.7; 46.8) | 29.3 | (24.3; 35.1) |
| 1988-89 | 35.0 | (26.5; 45) | 35.9 | (30.4; 41.8) | 36.8 | (28.5; 46.4) |
| 1990-91 | 49.4 | (37.9; 63.1) | 30.4 | (25.3; 37.1) | 25.4 | (20.3; 30.1) |
| 1992-93 | 46.4 | (33.9; 61.4) | 32.8 | (25.3; 40.4) | 30.0 | (24.1; 36.3) |
| 1994-95 | 38.4 | (25.8; 53.8) | 30.9 | (22.5; 41.3) | 29.3 | (23.1; 35.9) |
| 1996-97 | 30.6 | (18.7; 44.8) | 31.0 | (22.7; 41.5) | 22.0 | (17.1; 27.5) |
| 1998-99 | 30.6 | (19.1; 44.6) | 27.6 | (20.5; 35.2) | 22.8 | (16.9; 29.5) |
| 2000-01 | 47.0 | (23.8; 73.9) | 36.5 | (24.9; 49.5) | 19.4 | (14.7; 24.8) |
| 2002-03 | 36.9 | (13.2; 61.2) | 33.6 | (21.6; 46.6) | 22.6 | (16.9; 31) |
| 2004-05 | 32.4 | (14.9; 56.6) | 24.4 | (14.9; 34.9) | 29.4 | (16.8; 42.7) |
| 2006-07 | 9.7 | (3.8; 27.4) | 30.6 | (17.7; 46.1) | 24.4 | (15.5; 37.6) |
| 2008-09 | 33.0 | (12.3; 66.4) | 35.4 | (9.9; 73.6) | 23.3 | (14.4; 33.9) |
| 2010-11 | 13.7 | (6.5; 43.9) | 32.0 | (14.3; 60.8) | 14.6 | (6.9; 24.5) |
| Trend [*p*-value] | -1.57 | [0.001] | -0.60 | [0.020] | -1.10 | [0.000] |
|  |  |  |  |  |  |  |
|  | Some secondary or higher | |  |  |  |  |
| 1980-81 | 27.6 | (21.9; 34.5) |  |  |  |  |
| 1982-83 | 18.1 | (14; 22.5) |  |  |  |  |
| 1984-85 | 20.4 | (15.8; 25.9) |  |  |  |  |
| 1986-87 | 19.6 | (15.9; 23.6) |  |  |  |  |
| 1988-89 | 20.8 | (16.6; 25.1) |  |  |  |  |
| 1990-91 | 21.6 | (16.9; 27.7) |  |  |  |  |
| 1992-93 | 20.3 | (15.8; 24.9) |  |  |  |  |
| 1994-95 | 21.3 | (17.6; 26.1) |  |  |  |  |
| 1996-97 | 28.6 | (12.4; 51.8) |  |  |  |  |
| 1998-99 | 19.0 | (13.7; 25.9) |  |  |  |  |
| 2000-01 | 19.8 | (15.6; 24.8) |  |  |  |  |
| 2002-03 | 17.0 | (12.3; 21.9) |  |  |  |  |
| 2004-05 | 17.7 | (13.4; 22.1) |  |  |  |  |
| 2006-07 | 11.7 | (8.2; 16.1) |  |  |  |  |
| 2008-09 | 18.3 | (12.5; 24.2) |  |  |  |  |
| 2010-11 | 12.4 | (8.7; 17) |  |  |  |  |
| Trend [*p*-value] | -0.54 | [0.004] |  |  |  |  |
|  |  |  |  |  |  |  |
| **Island** | Java & Bali | | Sumatra | | Kalimantan, Sulawesi & NTMP | |
| 1980-81 | 40.2 | (35.5; 45) | 38.8 | (33.8; 43.9) | 43.2 | (38; 48.4) |
| 1982-83 | 34.6 | (30.1; 38.8) | 31.8 | (28.1; 35.9) | 39.2 | (34.4; 43.8) |
| 1984-85 | 35.2 | (30.3; 40.3) | 26.7 | (23; 31) | 32.2 | (27.5; 37.2) |
| 1986-87 | 35.1 | (29.8; 40.7) | 29.9 | (26.2; 34) | 33.6 | (29.7; 38) |
| 1988-89 | 37.4 | (30.2; 45.5) | 25.8 | (22.5; 29.6) | 34.1 | (29.9; 38.4) |
| 1990-91 | 27.7 | (23.2; 32.5) | 29.3 | (25.1; 33.5) | 29.4 | (25.6; 33.8) |
| 1992-93 | 27.8 | (23.4; 33.2) | 28.6 | (23; 34.1) | 32.2 | (28.1; 36.8) |
| 1994-95 | 30.4 | (23.4; 38.3) | 26.0 | (21.5; 30.8) | 26.4 | (22.5; 30.8) |
| 1996-97 | 22.3 | (15.3; 29.2) | 30.2 | (22.1; 40.6) | 25.8 | (21.5; 29.7) |
| 1998-99 | 20.7 | (15.5; 28) | 22.2 | (18.4; 26.6) | 26.5 | (22.2; 31.4) |
| 2000-01 | 20.9 | (15.6; 26.7) | 27.3 | (22.7; 32.5) | 25.4 | (19.4; 32.1) |
| 2002-03 | 19.3 | (13.9; 25.5) | 21.8 | (14.8; 29.8) | 27.0 | (21.9; 33) |
| 2004-05 | 17.2 | (10; 26.8) | 27.4 | (19.9; 34.9) | 29.1 | (22.5; 36.7) |
| 2006-07 | 16.0 | (10.8; 23.9) | 15.3 | (11.1; 20.2) | 20.3 | (14.5; 26) |
| 2008-09 | 19.4 | (11.6; 28.9) | 24.9 | (17.3; 33.3) | 23.1 | (16.4; 30.9) |
| 2010-11 | 13.8 | (8.4; 20.3) | 15.7 | (10.1; 22.7) | 14.7 | (8.5; 21.2) |
| Trend [*p*-value] | -1.70 | [0.000] | -0.94 | [0.000] | -1.32 | [0.000] |

*Notes*: NMR, neonatal mortality rate; CI, confidence interval; RR, rate ratio; RD, rate difference; Quin., Quintile; NTMP, Nusa Tenggara, Maluku and Papua. The small number of observations and possible non-linear relationships implies that the trend estimates should be treated with caution.
